# Novel CT Features of an Abdominal Gossypiboma in a Female Dog

**DOI:** 10.1155/2019/2865484

**Published:** 2019-06-24

**Authors:** Mylene Auger, Shelly Olin, Federica Morandi

**Affiliations:** Department of Small Animal Clinical Sciences, College of Veterinary Medicine, University of Tennessee, Knoxville, TN 37996, USA

## Abstract

**Case Description:**

An eight-year-old Golden Retriever was presented with hypercalcemia and a complex abdominal mass.

**Clinical Findings:**

A well-defined, heterogeneously contrast-enhancing, cavitary, soft tissue attenuating, non-organ associated abdominal mass was identified caudal to the right kidney. This mass was confluent with the distal tip of the right limb of the pancreas. A connected large, rim enhancing, cystic subcutaneous mass was also present in the right lumbar subcutaneous tissues, and there were multiple fistulous tracts through the hypaxial musculature.

**Treatment and Outcome:**

The dog underwent a surgical exploratory laparotomy and a gossypiboma was removed from the intra-abdominal mass; the cystic extra-abdominal mass was drained percutaneously. Surgical recovery was uneventful and a follow-up CT after 3 months was consistent with resolving granulomatous inflammation and fibrosis.

**Clinical Relevance:**

The presence of multiple peripherally enhancing tracts extending from the abdominal mass through the hypaxial musculature is a CT feature which has not yet been described in the veterinary literature. Additionally, incorporation of the pancreas into the abdominal mass has also not yet been described in the veterinary literature.

## 1. Introduction

Retained surgical sponges, also known as gossypibomas, are uncommonly diagnosed in veterinary medicine and the diagnosis can be challenging [1]. Radiographic and ultrasonographic features of gossypibomas have been reported in the veterinary literature; however, very limited information exists concerning their computed tomography (CT) characteristics [1–5]. The following report describes the clinical and diagnostic imaging features of an abdominal gossypiboma in a female dog, as well as CT features not previously reported in the veterinary literature. Knowledge of these features can aid in the diagnosis and treatment of this condition.

## 2. Case Description

An eight-year-old, spayed female Golden Retriever weighing 32 kg was presented to the University of Tennessee Veterinary Medical Center (UT-VMC) for evaluation of an intra-abdominal mass and hypercalcemia. The dog was spayed 6 years previously and reportedly normal until 10 days prior to presentation, when the dog was evaluated by the referring veterinarian for lethargy. A large cystic mass measuring 10 cm x 7 cm x 3 cm was noted on the right flank, which was drained by the referring veterinarian. This mass had reportedly been present for approximately one year prior to presentation. The dog was treated empirically with cephalexin at 22 mg/kg PO q12h (AmerisourceBergen, Chesterbrook, PA, USA). The lethargy resolved, and the dog was clinically normal at the time of presentation to UT-VMC, aside from reportedly licking and biting at its right flank. Physical examination revealed persistence of the reported subcutaneous cystic mass on the right flank as well as a firm, nonpainful intra-abdominal mass caudal to the right kidney.

Hematology was unremarkable. Biochemistry revealed mild total hypercalcemia (13.3mg/dL; reference range 10-12 mg/dL), normal phosphorus (2.8 mg/dL; reference range 2.5-5.9 mg/dL), mildly elevated creatinine (1.3 mg/dL; reference range 0.3-1.1 mg/dL), normal BUN (16 mg/dL; reference range 7-37 mg/dL), and mild hyperglobulinemia (4.1 g/dL; reference range 1.9-3.1 g/dL). Urinalysis revealed isosthenuria but was otherwise unremarkable. A hypercalcemia of malignancy profile was performed at the Michigan State University Veterinary Diagnostic Laboratory where these tests had previously been validated, revealing marked ionized hypercalcemia (1.75 mmol/L; reference range 1.26-1.39 mmol/L), a plasma PTH concentration below the reference range (0 pmol/L; reference range 0.5-5.8 pmol/L), and normal plasma PTHrP concentration (0 pmol/L; reference range 0.0-1.0 pmol/L). Serum concentration of 1,25[OH]2D was normal (97 nmol/L; reference range 60-125 nmol/L).

**Figure 1:**
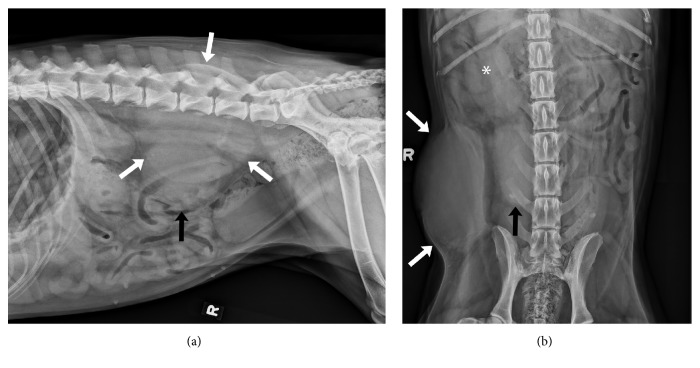
(a) Right lateral and (b) ventrodorsal radiographs of the caudal portion of the abdomen. A large, well-defined, ovoid, soft tissue opaque mass is seen in the right caudal abdominal subcutaneous tissues (white arrows). A large, relatively well-defined, soft tissue opaque mass is noted in the right caudal abdominal cavity (black arrow), caudal to the right kidney (*∗*).

Radiographic (Super 80CP, Philips Medical Systems, Bothell, WA) findings included two large right caudodorsal abdominal soft tissue opaque masses in close proximity to one another, with evidence of mild fluid streaking of the fat surrounding both structures (Figure 1). The first mass was located within the subcutaneous tissues of the right caudodorsal abdominal wall and was associated with focal medial deviation of the abdominal wall. The second was within the right caudodorsal abdominal cavity, caudal to the right kidney. It could not be determined whether the abdominal mass was associated with the body wall mass or whether these represented two distinct processes. Differential diagnoses for the intraabdominal mass included a granuloma, hematoma, or neoplasia, possibly originating from the mesentery or a regional lymph node. Differential diagnoses for the abdominal wall mass included benign or malignant etiologies such as a granuloma, abscess, or a sarcoma.

**Figure 2:**
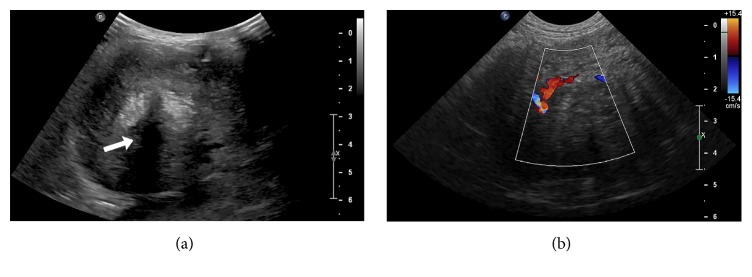
(a) Longitudinal plane sonographic image displaying a well-defined, heterogeneous, non-organ associated abdominal mass with strongly hyperechoic foci within its center, associated with acoustic shadowing (white arrow). (b) Longitudinal plane sonographic image displaying the vascularity of the abdominal mass when interrogated with color Doppler.

Abdominal ultrasound (Epiq 5, Philips Ultrasound, Bothell, WA, USA) was performed next to further characterize the identified masses. The sonographic examination was performed with the patient in dorsal recumbency using a microconvex 8 MHz transducer, a convex 9 MHz transducer, and a linear 12 MHz transducer. A heterogeneous, non-organ associated abdominal mass with strongly hyperechoic, shadowing foci within its center was identified in the right caudal abdomen (Figure 2(a)). This mass had multiple finger-like hyperechoic projections extending laterally and caudally, connecting it with the large extra-abdominal, cystic mass in the right lumbar region. The intra-abdominal mass was moderately vascularized when interrogated with color Doppler (Figure 2(b)), with evidence of multiple relatively large, slightly tortuous, branching intralesional blood vessels. Given the sonographic appearance of these lesions, differential diagnoses included malignant neoplasia, such as sarcoma or carcinoma, or a granulomatous inflammatory process (such as secondary to a chronic foreign body or fungal infection). Ultrasound-guided fine-needle aspiration of the abdominal mass was performed to obtain tissue samples for cytologic analysis, which was consistent with pyogranulomatous inflammation.

**Figure 3:**
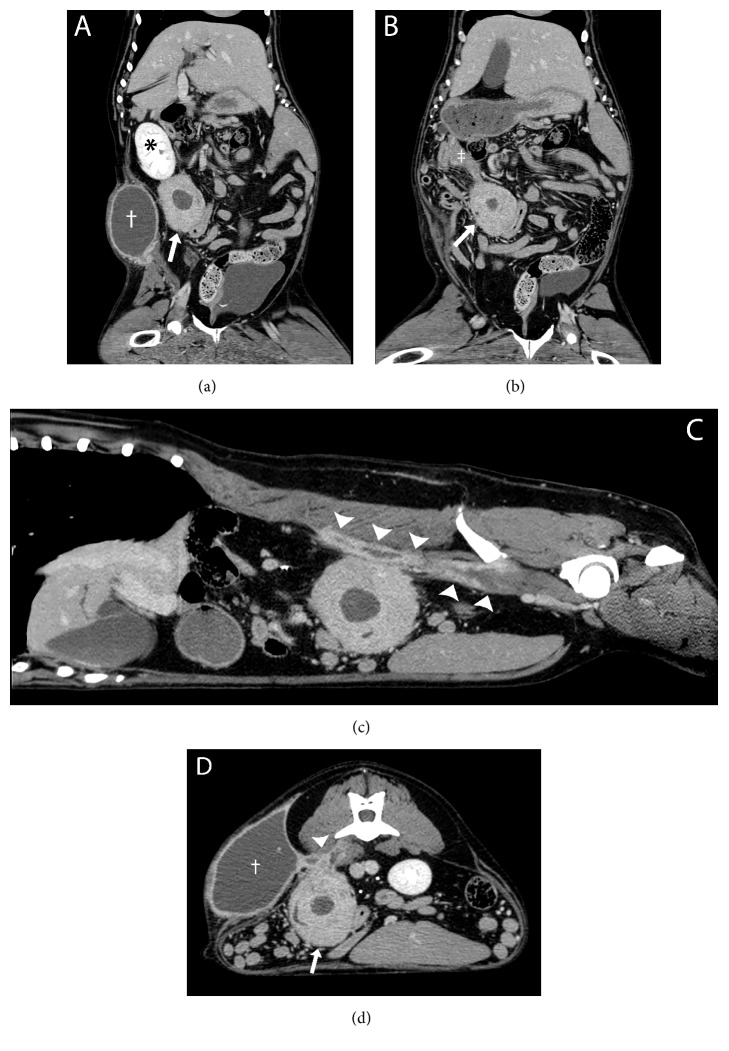
Postcontrast computed tomographic images of the abdomen on the patient's initial presentation, displayed in soft tissue window. (a) Dorsal oblique multiplanar reconstruction displaying a large, heterogeneously contrast-enhancing, cavitary, non-organ associated abdominal mass (white arrow), caudal to the right kidney (*∗*). A large, rim enhancing, cystic subcutaneous mass (†) is also noted in the right lumbar subcutaneous tissues, resulting in focal medial displacement of the abdominal wall. (b) Dorsal multiplanar reconstruction displaying the association between the abdominal mass (white arrow) and the right limb of the pancreas (‡). (c) Sagittal multiplanar reconstruction displaying the rim enhancing fistulous tracts (arrowheads) extending in a cranial to caudal fashion in the right hypaxial musculature. (d) Transverse image displaying the rim enhancing fistulous tracts (arrowhead) interconnecting the abdominal mass (white arrow), cystic subcutaneous mass (†), and hypaxial musculature.

CT (Brilliance, Philips Medical Systems, Cleveland, OH, USA) of the abdomen was performed using a 40-slice helical scanner for further characterization of the relationship between the intra-abdominal and extra-abdominal lesions, in preparation for surgical excision. A submillimeter dataset of the abdomen was acquired and images were reconstructed in 0.9mm, 1.5 mm, and 5 mm slice thickness utilizing bone and soft tissue algorithms. The acquisition was repeated following intravenous administration of Ioversol 350 mgI/ml, a nonionic iodinated contrast medium (Tyco Healthcare/Mallinckrodt, Milwaukee, WI, USA) at a dosage of 2.2 mg/kg IV. A well-defined, heterogeneously contrast-enhancing, thick-walled, cavitary, soft tissue attenuating, abdominal mass was present caudal to the right kidney (Figure 3(a)). This mass was confluent with the distal tip of the right limb of the pancreas (Figure 3(b)) and intimately associated with a small intestinal segment. Despite its close association with the small intestine and pancreas, this mass was not centered on these structures and therefore was most consistent with a non-organ associated abdominal mass with secondary involvement of adjacent abdominal organs. A few pinpoint mineral attenuating foci were noted within this mass. A large, rim enhancing, cystic subcutaneous mass was also identified in the right lumbar subcutaneous tissues, resulting in focal medial displacement of the abdominal wall (Figure 3(a)). In addition, ill-defined, peripherally contrast-enhancing tracts were seen extending through the right hypaxial musculature from the level of the midbody of the L4 vertebra to the level of S3 (Figure 3(c)). Both masses and the peripherally contrast-enhancing tracts in the hypaxial musculature were all interconnected by thick, peripherally contrast-enhancing soft tissue attenuating stalks (Figure 3(d)). Given the confirmed connection of the two masses, extra-abdominal extension of an intra-abdominal mass with multiple fistulous tracts led to a primary differential diagnosis of a granulomatous inflammatory process, such as secondary to a chronic foreign body. An aggressive soft tissue neoplasm (i.e., soft tissue sarcoma) was also considered.

An exploratory laparotomy was subsequently performed. The intra-abdominal portion of the mass was large (7.5 cm x 4.5 cm) and nonresectable because it was highly vascularized with extensive adhesions to the colon and omentum. The thick, fibrous capsule was incised to reveal fluid and a 4-inch x 4-inch gauze, most likely from the ovariohysterectomy 6 years earlier. A wedge biopsy of the mass was obtained for histopathological analysis and culture. The mass was lavaged, omentalized, and sutured closed. The abdomen was flushed with sterile saline and closed. A stab incision was made into the second subcutaneous cystic mass dorsolateral to the incision site. The content of this cystic mass was drained using suction. A Jackson-Pratt drain (Cardinal Health, Waukegan, Illinois, USA) was subsequently placed into this structure and secured in place for 24 hours. Approximately 240 mL of fluid was drained from the extra-abdominal portion of the mass.

Histopathology of the biopsy sample from the intraabdominal mass revealed chronic, fibrosing, pyogranulomatous and lymphoplasmacytic fasciitis with marked pancreatic atrophy and loss. This was consistent with a gossypiboma which had incorporated and replaced portions of the pancreas, likely the most distal aspect of the right pancreatic limb which was intimately associated with the mass on presurgical imaging. Aerobic and anaerobic culture of a portion of the biopsy sample revealed* Streptococcus agalactiae* from broth only, and 2 colonies of* Staphylococcus* sp. No growth was seen on fungal culture after 5 weeks.

Postoperatively, the dog was treated supportively with intravenous fluid therapy, a fentanyl/lidocaine constant rate infusion (AmerisourceBergen, Chesterbrook, PA, USA) at a dosage varying between 2 and 5 mcg/kg/hr, ampicillin (AmerisourceBergen, Chesterbrook, PA, USA) (705 mg, IV, q 8 h), and zoledronate (Novartis Pharmaceuticals Corp, East Hanover, NJ) at a total dosage of 4 mg IV given once. Ionized calcium normalized within 48 hours (1.34 mmol/L; reference range 1.26-1.39 mmol/L). The dog recovered uneventfully and was discharged 24 hours following surgery with tramadol (AmerisourceBergen, Chesterbrook, PA, USA ) at a dosage of 3 mg/kg PO q12h for 5 days, and amoxicillin (Zoetis, Parsippany, NJ, USA) at a dosage of 24 mg/kg PO q 12 h for 10 days.

**Figure 4:**
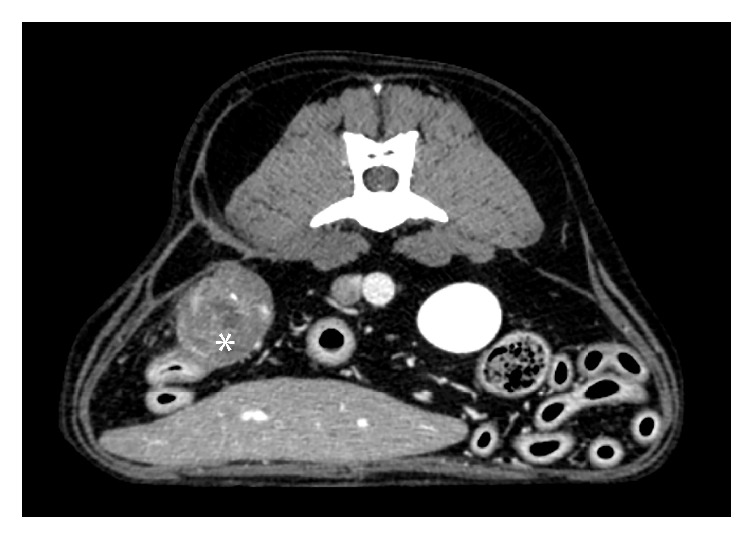
Transverse postcontrast computed tomographic image approximately three months following surgical debridement of a gossypiboma, displayed in a soft tissue window. The soft tissue attenuating mass within the right caudal abdomen (*∗*) has markedly decreased in size and is less contrast-enhancing than prior to surgery with persistence of a non-contrast-enhancing hypoattenuating center. The previously seen large, cystic subcutaneous mass is no longer present.

The dog was reevaluated approximately 3 months following surgery. Clinically the dog was normal and normocalcemic (ionized calcium 1.32 mmol/L; reference range 1.26-1.39 mmol/L). Abdominal CT was repeated prior to and following intravenous contrast medium administration using the same imaging parameters as the initial examination. The soft tissue attenuating mass within the right caudal abdomen had markedly decreased in size and was less contrast-enhancing, with persistence of a non-contrast-enhancing hypoattenuating center (Figure 4), and few pinpoint, mineral attenuating foci within it. The thick stalk previously extending from the mass through the right abdominal wall was no longer identified, and the stalk connecting the abdominal mass to the hypaxial musculature was decreased in thickness and no longer contrast-enhancing. There was however persistence of a thin, non-contrast-enhancing, soft tissue attenuating stalk connecting the cranial aspect of this mass to the distal tip of the right limb of the pancreas. The large cystic mass in the right dorsolateral abdominal subcutaneous tissues had resolved, with only a small, ill-defined region of mildly contrast-enhancing thickening of the subcutaneous tissues remaining. The fistulous tracts extending through the right hypaxial musculature had resolved. These findings were consistent with resolving granulomatous inflammation following surgical debridement of a gossypiboma. The residual stalks interconnecting some of these structures and the ill-defined, mild thickening of the right midabdominal subcutaneous tissues were most consistent with residual fibrosis, although persistence or progression of previously resolved fistulous tracts could not be entirely excluded due to lack of additional follow-up imaging.

## 3. Discussion

Radiographic features of retained surgical sponges have been described in the veterinary and human literature. Certain surgical sponges have radiopaque markers, which facilitate their radiographic identification [2]. In the case of nonradiopaque sponges, the most commonly described feature is a focal gas lucency which has a speckled or whirl-like appearance [2, 3, 5]. This feature was not identified in our case, justifying additional imaging for further evaluation. Additional described radiographic features include intestinal obstruction, a mass or peripheral mineralization [2].

The reported sonographic features of gossypibomas in dogs are a well-defined hypoechoic mass with an irregular, hyperechoic center which often contains strongly hyperechoic, shadowing foci [2, 3, 5]. These features are consistent with the ultrasound features of the abdominal mass in our patient; however, the presence of multiple hyperechoic projections extending from this mass, at least one of which connected it to a large subcutaneous cystic mass, justified the use of CT to determine the exact extent of organ involvement.

Very little information is available concerning the CT characteristics of gossypibomas in veterinary medicine. A recent report of spontaneous transmural migration of a retained surgical sponge into the cecum of a dog has described the CT appearance of the gossypiboma as a heterogeneous mass with a speckled or spongiform gas pattern [4]. The spongiform pattern was thought to result from gas trapped within the retained sponge [4]. Similar features have been described in the human literature, where CT is the preferred imaging modality for evaluation of retained surgical sponges [4]. In humans, the commonly reported CT features of gossypibomas are a heterogeneous mass with a contrast-enhancing wall and a spongiform pattern [2, 4]. Mineral attenuating foci within the mass or the presence of radiopaque markers within the mass have also occasionally been described [4]. Although the classic spongiform pattern was not identified in our patient, the presence of a non-organ associated abdominal mass with a thick contrast-enhancing rim was consistent with the reported CT features of gossypibomas in humans. Furthermore, CT was useful in characterizing the interrelationships between the subcutaneous and abdominal masses as well as identifying involvement of the pancreas. We also describe the presence of multiple peripherally enhancing tracts extending from the abdominal mass through the hypaxial musculature, a CT feature which has not yet been described in the veterinary literature.

A recent retrospective study evaluating the clinical features of 13 dogs with retained surgical sponges identified 4 cases which presented with chronic fistulous tracts, 3 of which were in the flank, similar to the location of our patient's cystic subcutaneous mass [1]. In cases involving the flank in this study, the original surgery was ovariohysterectomy, as was the case with our patient [1]. In one report of a dog with an enterocutaneous fistula secondary to a retained surgical swab, a subcutaneous mass was present in the flank which subsequently ruptured, leading to a chronic fistula [6]. It is possible that the subcutaneous mass in our patient would have eventually ruptured, leading to the more common presentation of a chronic fistulous tract.

More serious complications associated with retained surgical swabs include osteomyelitis, infiltration of the gauze into the urinary bladder mimicking bladder neoplasia, a retained surgical sponge mimicking an implant-associated sarcoma, malignant transformation, enterocutaneous fistula, and intestinal obstruction following transmural migration [1, 4–8]. In the present case, there was incorporation of the pancreas into the abdominal mass, a feature which, to our knowledge, has not yet been described in the veterinary literature. Furthermore, although fistulous tracts through the flank have been reported with abdominal gossypibomas, fistulous tracts extending through the hypaxial musculature, as were seen in our patient, have also not yet been described in the veterinary literature.

The most common causes of hypercalcemia in dogs are malignancy, hypoadrenocorticism, primary hyperparathyroidism, and chronic kidney disease [9]. Less common causes include hypervitaminosis D, granulomatous diseases, dehydration, and laboratory error [9]. At presentation, the patient's bloodwork showed total and ionized hypercalcemia with low parathyroid hormone (PTH), absent (or normal) parathyroid hormone-related protein (PTHrP), and normal 25-hydroxyvitamin-D levels. These results excluded primary hyperparathyroidism and hypervitaminosis D as causes of the hypercalcemia. The normal parathyroid hormone-related protein (PTHrP) result decreased the likelihood for a diagnosis of hypercalcemia of malignancy. Granulomatous inflammation usually results in elevations in calcium with low values of PTH [10]. Hypercalcemia associated with granulomatous diseases is thought to be due to either an alteration of endogenous vitamin D metabolism due to activation of macrophages, resulting in an excessive production of 1,25(OH)2D, or due to synthesis of PTHrP, both of which were normal in our patient [9, 10]. A granulomatous inflammatory process was nonetheless the most likely cause of our patient's hypercalcemia when considering the results of abdominal exploratory surgery, histopathology, and normalization of serum calcium following surgical and medical therapy. The underlying mechanism of our patient's hypercalcemia is however not completely known.

## 4. Conclusion

In conclusion, this report describes CT characteristics of an abdominal gossypiboma which have not yet been reported in the veterinary literature, including multiple peripherally enhancing tracts extending from the abdominal mass through the hypaxial musculature and incorporation of the pancreas into the abdominal mass. In association with the large cystic mass in the right flank, such features should raise suspicion for a retained surgical sponge, which carries a good prognosis when surgically addressed, as was the case with the patient in this case.
